# Aflatoxin B_1_ in Affecting Broiler’s Performance, Immunity, and Gastrointestinal Tract: A Review of History and Contemporary Issues

**DOI:** 10.3390/toxins3060566

**Published:** 2011-06-14

**Authors:** Agha W. Yunus, E. Razzazi-Fazeli, Josef Bohm

**Affiliations:** 1 Institute of Animal Nutrition, University of Veterinary Medicine Vienna, A-1210 Vienna, Austria; Email: Josef.Boehm@vetmeduni.ac.at; 2 VetCore Facility for Research, Proteomics Unit, University of Veterinary Medicine Vienna, A-1210 Vienna, Austria; Email: ebrahim.razzazi@vetmeduni.ac.at

**Keywords:** aflatoxin, broiler, chicken, hormesis

## Abstract

Aflatoxin B_1_ is a common contaminant of poultry feeds in tropical and subtropical climates. Research during the last five decades has well established the negative effects of the mycotoxin on health of poultry. However, the last ten years of relevant data have accentuated the potential of low levels of aflatoxin B_1_ to deteriorate broiler performance. In this regard, any attempt to establish a dose-effect relationship between aflatoxin B_1_ level and broiler performance is also complicated due to differences in types of broilers and length of exposure to the mycotoxin in different studies. Contrary to the prevalent notion regarding literature saturation with respect to aflatoxicosis of chicken, many areas of aflatoxicosis still need to be explored. Literature regarding effects of the mycotoxin on the gastrointestinal tract in this regard is particular scanty and non-conclusive. In addition to these issues, the metabolism of aflatoxin B_1_ and recently proposed hypotheses regarding biphasic effects of the mycotoxin in broilers are briefly discussed.

## 1. Introduction

**Figure 1:**
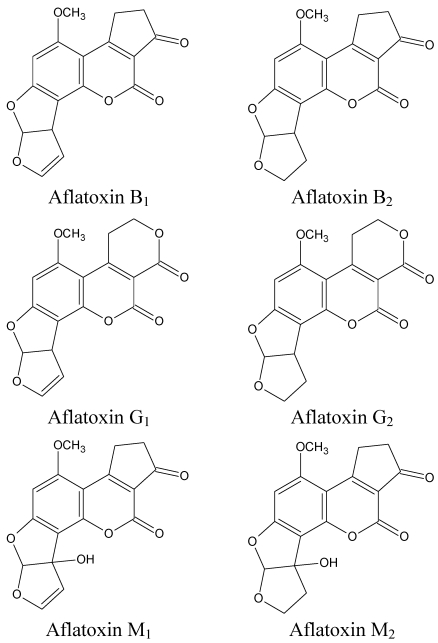
Structure of aflatoxins.

Aflatoxins, secondary metabolites of various *Aspergillus* spp., commonly contaminate a wide variety of tropical and subtropical food/feed stuffs. These mycotoxins are known to have strong hepatotoxic and carcinogenic effects and are regulated by feed/food law in at least 100 countries [1]. Chemically, aflatoxins are difuranocoumarin compounds and include B_1_, B_2_, G_1_, G_2_, M_1_, and M_2_ [2] (Figure 1). These mycotoxins contaminate a wide variety of agricultural commodities including oilseed meals, dried fruits, spices, and cereals [3]. Aflatoxins M_1_ and M_2_ however, mainly occur in milk (AFM_1_ in small quantities also reported in eggs) as metabolites of the B_1_ and B_2_. Among the various types of aflatoxins, aflatoxin B_1_ (AFB_1_) is most commonly encountered and it is also considered to have higher toxicity than other aflatoxins.

The discovery and isolation of aflatoxins is well known to be a result of investigations on the mysterious Turkey-X disease of 1960 which resulted in loss of several thousand turkey poults in the United Kingdom. The cause of enormous mortality in turkey poults and of similar outbreaks in other farm animals could be linked with the use of moldy Brazilian peanut meal in the diet of affected animals [4]. The suspected toxic factor was found to be extractable by using chloroform [5]. Its association with *Aspergillus flavus* could then be established in the year 1961 [6]. In 1962, the name “aflatoxin”, using first letter from “Aspergillus” and the first 3 letters from “flavus” was proposed [7]. Aflatoxin was in the same year isolated in crystalline form in the Netherlands [8], and separated into two components, B and G in the United Kingdom [9]. This was followed by a further division of the aflatoxin B into B_1_ and B_2_ and later their chemical characterization by Asao *et al.* [10]. Details of these landmarks and other studies have been reported in earlier reviews [7,11].

Since the discovery of aflatoxins, their negative effects on animal health have been an active area of research. In this regard, research during the last five decades has well elucidated the negative effects of aflatoxins on animal performance and immunity. To date, various aspects of the aflatoxicosis in farm animals including effects on animal performance and metabolism, metabolism of the toxin, and carryover of toxic residues to animal products have been the subjects of several comprehensive reviews [12]. However, some aspects of aflatoxicosis, particularly effects on gastrointestinal tract (GIT), are not well documented. The present review therefore intends to encompass these areas of aflatoxicosis in broilers. Furthermore, contemporary issues regarding dose-effect relationship between dietary levels of AFB_1_ and broiler performance [13], and recently proposed biphasic effects of the toxin on broiler’s weight gain [14] are discussed. The latter hypothesis regarding aflatoxicosis is extended to other variables of broiler health wherever sufficient data are available.

## 2. Metabolism of Aflatoxin B_1_

Relative sensitivity of various animal species to AFB_1_ has been presented in Table 1. The sensitivity of chicken is comparative to that of rats, and both species appear to be insensitive on a comparative scale. The difference regarding sensitivity of various animal species towards AFB_1_ is thought to be linked with differential state of the toxin’s metabolism and the types of metabolites formed [15]. However, many aspects of metabolism of AFB_1_ in chickens need to be investigated.

**Table 1:** Comparison of LD_50_ and acute effects of AFB_1_ on liver of various animal species ^1^.

Species	LD_50_	Lesions in Liver					
		Necrosis and Hemorrhage	Fibrosis	Regeneration of Nodules	Bile Duct Proliferation/Hyperplasia	Vacuolation and Fatty Infiltration	Enlarged Hepatic Cells
Rabbit	0.4	+	-	+	+	-	+
Duckling	2.8	+	-	+	+	+	+
Pig	3.9	+	+	+	+	+	+
Dog	6.3	+	+	+	+	+	+
Guinea pig	10.6	+	-	+	+	+	+
Sheep ^2^	12.5						
Mouse	56.3	-	-	-	-	+	+
Chicken	72.0	-	-	-	+	+	+
Rat	73.3	+	-	+	+	+	+

^1^ Modified from Patterson [7], with data on chicken from Miazzo *et al.* [16], and Denli *et al.* [17]. LD_50_ in mg/kg body weight. ^2^ Data not available, however metabolism of AFB_1_ is slower in sheep [15]. Sufficient data indicate reduced weight of liver [18], and hepatic carcinoma [19] in sheep. Abbrev.: + noted effects; - effects not noted; empty cells indicate lack of data.

### 2.1. Absorption and Excretion

Work done utilizing murine models indicate that absorption of aflatoxins is a very fast process that follows first order kinetics [20,21]. Approximately all of the orally administered AFB_1_ has been noted to be absorbed in rats [22,23]. Absorption is followed by an extensive transformation into metabolites primarily in liver [24]. However, the elimination of aflatoxins from body is slower as compared to the case of other mycotoxins especially trichothecenes. Wong and Hsieh [25] investigated the excretion of ^14^C-labelled AFB_1_ in mice, rats, and monkeys. These authors found the excretion of AFB_1_ to be high during initial 24 h of the i.v. injection. However, the total recovery of the administered AFB_1_ was between 72 and 80% during the first 100 h after the i.v. injection. In case of laying hens, 71% of the ^14^C-labelled AFB_1_ administered into crop could be recovered within 7 days post-administration [26]. In this study, only 28% of the administered dose of AFB_1_ could be recovered during first 24 h. On day 1, 4, and 7 of post-administration of ^14^C-labelled AFB_1_, the accumulation of radioactivity was estimated by these authors to be 1.3, 1, and 1.1% of the total administered dose. Liver and reproductive organs were found to be the main sites for accumulation of radioactivity. In a contemporary study, Mabee and Chipley [27] investigated the metabolism of AFB_1_ during continuous exposure. These authors administered ^14^C-labelled AFB_1_ to laying hen by using crop intubation tube for 14 consecutive days. At 5 h post-intubation of the last dose, the total radioactivity in hens was approximately equal to the daily dose of the toxin. It was therefore concluded that most of the ^14^C-labelled AFB_1_ administered during first 13 days was excreted before administration of the final dose on 14th day—providing a clue that elimination AFB_1_ is faster during continuous exposure. Wolzak *et al.* [28] have reported that tissue residues of aflatoxins were highest in kidney, gizzard, and liver (average conc. 3 μg/kg mass) when broilers were exposed for 4 weeks to a mixture of AFB_1_ and AFB_2_. After 7 days of removal of the contaminated feed, aflatoxin residues could not be detected in aforementioned tissues. In this regard, a recent study by Hussain *et al.* also indicates that the elimination of AFB_1_ in chicken increase during longer exposure to AFB_1_ [29]. These authors fed broiler chicks on rations containing 0, 1.6, 3.2, and 6.4 µg AFB_1_/kg for 7, 14, or 28 days age. After 2 to 3 days of exposure, AFB_1_ could be detected in livers of the birds exposed to 1.6 µg AFB_1_/kg and higher dietary levels of the toxin. After cessation of toxin feeding, AFB_1_ residues decreased in livers and muscles of all the birds, with lower levels at 10 days post-cessation in the birds exposed to higher toxin levels. These authors concluded that the residues of AFB_1_ in tissues increase with increase in dietary concentration of the toxin but decrease with increase in age (or after longer exposure) of broiler chicks. The elimination of AFB_1_ from tissues was rapid in older birds than in younger birds.

### 2.2. Metabolism

**Figure 2:**
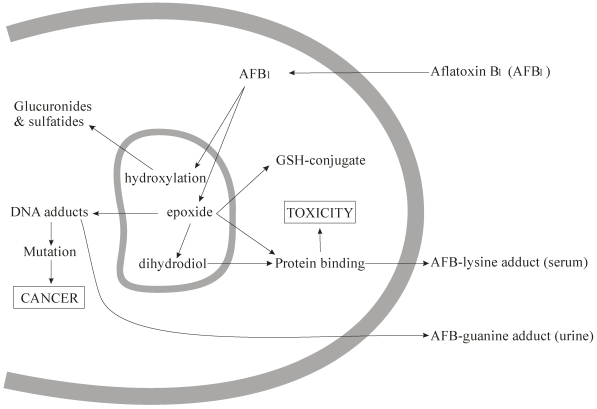
Mechanisms of AFB_1_ toxicity [32]. In the endoplasmic reticulum, AFB_1_ is converted to hydroxylated metabolites (via monooxygenases) which are then metabolized to glucuronide and sulfate conjugates. An alternate pathway is the oxidation of AFB_1_ to form AFB_1_-8,9-epoxide which can further undergo hydrolysis to form AFB_1_-8,9-dihydrodial. The epoxide can also be conjugated (to form GSH-conjugate) and thus detoxified by glutathione *S*-transferases.

Besides being the primary organ of AFB_1_ accumulation and metabolism, liver is also the main site where AFB_1_ is metabolized and where the metabolites bind with nucleic acids and proteins. Kidneys also take part in detoxification of aflatoxins and are also among the organs where most of the aflatoxin residues are detected [26,30]. The metabolism of AFB_1_ after absorption has been previously reviewed in detail [7,24,31,32,33,34,35]. In summary, cytochrome P450 enzymes (CYP) (including CYP1A2, CYP3A4 and CYP2A6) in the liver and other tissues convert AFB_1_ to epoxides (AFB_1_-8,9-exo-epoxide, and AFB_1_-8,9-endo-epoxide), and to AFM_1_, AFP_1_, AFQ_1_, and its reduced form aflatoxicol (Figure 2). Of the epoxides, the AFB_1_-8,9-exo-epoxide (and not the AFB_1_-8,9-endo-epoxide) can form covalent bonds with DNA and serum albumin resulting in AFB_1_-N7-guanine and lysine adducts, respectively. Like AFB_1_, AFM_1_ can also be activated to form AFM_1_-8,9-epoxide that binds to DNA resulting in AFM_1_-N7-guanine adducts. These guanine and lysine adducts have been noted to appear in urine. The metabolites AFP_1_, AFQ_1_, and aflatoxicol are thought to be inactive and are excreted as such in urine, or in the form of glucuronyl conjugates from bile in feces.

In case of chicken exposed to AFB_1_ contaminated rations, AFB_1_, AFM_1_, and aflatoxicol have been detected in liver, kidneys, and thigh muscles [36]. Besides these, AFB_2a_ has also been detected in livers of both broilers and layers on a ration contaminated with a mixture of aflatoxins (AFB_1_ 80%; AFB_2_ 2.6%; AFG_1_ 16.8%; and AFG_2_ 0.1%) [30]. Recent studies have shown that CYP2A6 and to a lesser extent YP1A1 are responsible for bio-activation of AFB_1_ into epoxide form in the liver of chicken and quail [37]. More data are however needed to fully understand the differences in metabolism of chicken with species which are comparatively more sensitive to AFB_1_.

## 3. Effects of Aflatoxin B_1_ on Performance and Serum Chemistry

Various reports on effects of aflatoxins on bird performance and serum chemistry have been previously reviewed by Patterson [7], Dersjant-Li *et al.* [13], and Devegowda and Murthy [38]. There is a general agreement that dietary aflatoxins reduce weight gain, feed intake, and increase feed conversion ratio. Information from the aforementioned reviews and some recent studies is summarized in Table 2. These data indicate that AFB_1_ has the capability to reduce broiler performance and increase the incidence of bruising in carcass when present at levels of more than 0.5 mg/kg diet. Dersjant-Li * et al.* in this regard concluded in their review that each mg of AFB_1_/kg diet would decrease the growth performance of broilers by 5% [13]. However, data published during last decade regarding effect of low doses of AFB_1_ on weight gain is not consistent with this generalization. For instance, Raju and Devegowda [39] noted 21% decrease in final body weight at 35 days age in broilers fed on 0.3 mg AFB_1_/kg diet. Contrary to this, Tedesco *et al.* [40] noted only 10% reduction in weight gain of broilers at 28 days of exposure to 0.8 mg AFB_1_/kg diet. For levels of AFB_1_ of 1 mg/kg diet, 10% reduction in weight gain was noted by Zhao * et al.* [41] at 21 days of exposure while 15% reduction at 42 days exposure was noted by Denli * et al.* [17]. At further higher levels of 3 mg AFB_1_/kg diet, only 11% reduction in weight gain at 21 days exposure was noted by Valdivia * et al.* [42]. Similarly, Miazzo * et al.* [16] found 11% reduction in weight gain when 2.5 mg AFB_1_/kg diet was fed to broilers from 21 to 42 days of age. From these reports, it is evident that both the level and length of AFB_1_ exposure affect the amount of reduction in weight gain of broilers. Furthermore, different type of and rations used in different studies make it impractical to generalize the dose-response relationship regarding weight gain.

**Table 2:** Summary of effects of AFB_1_ on gross performance variables in chicken.

AFB_1_ (mg/kg)	Performance *^,1,2,4^	Bruising ^3^	Weight of Organs				Serum ^1,3,4^	
			Liver ^1,4^	Spleen ^1,4^	Bursa and Thymus ^5^		Lipid	Protein
≤0.1	~		~				~	~
0.5	↓	~	~				~	~
1.0	↓	↑	↑		↓		↓	↓
2.5	↓	↑	↑	↑	↓		↓	↓
≥5.0	↓	↑	↑	↑			↓	↓

* Bird performance variables include body weight gain, feed consumption. Abbrev.: empty cells indicate lack of effect; ~ indicates inconsistent data; ↑ indicate increase; ↓ indicate decrease; ? indicates lack of data; enzyme activity in terms of lysosomal enzyme activity; TP, total protein; wt., weight. ^1^ [7]; ^2^ [13]; ^3^ [38]; ^4^ [16,17,39,40,41,42,43,44,45,46,47,48,]; ^5^ [49,50].

It is interesting to mention that many authors who reviewed the studies conducted prior to the 1980s considered 1.25 mg AFB_1_/kg diet as not having any negative effects on broiler performance [7]. Recent literature, as briefly reviewed in the preceding paragraph, on the other hand documents negative effects of lower levels of the toxin on broiler performance. Even the levels of AFB_1_ as low as 0.02 mg/kg diet have been indicated to decrease weight gain of broilers by 5% (*P* < 0.05) in a 3 weeks feeding study [48]. One explanation of these differences in earlier and recent reports could be the difference in the performance of broilers available at the time of study. Modern broiler in this connection is known to gain more weight by utilizing less feed in shorter time [51,52,53]. As AFB_1_ is known as hepatotoxic, it might result in more profound negative effects in birds with more efficient nutrient conversion demanding faster hepatic metabolism. Differences in the susceptibility of broilers and layers in this regard have been already postulated to be due to differences in metabolic rate of these bird types. Yet another possible cause of these differences could be sensitivity of analytical methods available at the time of previous and present studies.

In a recent review, Diaz *et al.* proposed that the effects of AFB_1_ on weight gain in broilers could be of biphasic nature (hormesis), *i.e.*, improvement at low doses while reduction at high doses [14]. In the review of Diaz *et al.*, the maximum improvement in weight gain of broilers was stated to be 3 to 4% during exposure to low levels of AFB_1_. In the aforementioned report of Tedesco *et al.* [40] these authors however noted 13% improvement in weight gain of broilers during 2nd week of exposure to 0.8 mg AFB_1_/kg diet. After 2nd week of exposure the weight gain of broilers started to decline under AFB_1_ diet with statistically significant effects apparent during 4th week of exposure. It therefore seems that the length of exposure to AFB_1_ besides its level could also influence the type of response regarding weight gain. However these improvements in weight gain, though might be of economic importance, were never reported to be of any statistical significance.

Studies conducted during last decade on effects of AFB_1_ on serum chemistry are summarized in Table 3. From the presented data, it is apparent that AFB_1_ at levels of up to 0.3 mg/kg decreases serum cholesterol levels. As the dietary level of AFB_1_ increases to 1 mg/kg, total serum protein and albumin contents are decreased. At further higher levels of 2 mg/kg diet, lower serum glucose, Ca, and inorganic P levels are recorded. Though Raju and Devegowda [39] reported lower total serum protein in broilers exposed to 0.3 mg AFB_1_/kg diet, several other authors including Tedesco *et al.* [40] could not note any effects of higher doses of AFB_1_ on this variable. From the presented data, it is also not possible to draw a dose-effect relationship for levels of serum enzymes including alkaline phosphatase, alanine transferase, γ-glutamyl transferase. However, altered concentrations of these enzymes are usually noted at 1 mg AFB_1_/kg diet. Besides these effects, AFB_1_ is also known to induce glutathione depletion and result in lipid peroxidation [54].

**Table 3:** Effect of AFB_1_ on hematology and serum chemistry, as noted in recent studies.

AFB_1_ Level (ppm)	*n* *	Hematology and Serum Chemistry		Year of Study and Reference
Bird Type, and Age (days)		Effects	No Effects	
0, 0.1	4	↓ AP	AST, γ-GT, TP, Chl, BUN, creatinine	2010 [55]
♂Ross308, 427–457	(12)			
0, 0.3	12	↓ TP and Chl at 21 days	BUN, ALT, γ-GT, AST at 21 days. BUN, ALT, Hb at 35 days	2000 [39]
Broilers, 1–35		↓ TP, Chl, γ-GT, AST at 35 days		
0, 0.8	7	↓ ALT	TP, albumin, globulin, Glc., AST, γ-GT, Ca, P	2004 [40]
♂Broilers, 14–49				
0, 1.0	4	↓ TP, albumin, Chl, Ca	Uric acid, γ-GT, P	2008 [47]
♂Cobb, 1–21	(8)			
0, 1.0	10	↑ AP	TP, albumin, AST, γ-GT, uric acid, Chl, triglyceride	2009 [17]
♂Ross308, 1–42				
0, 1.0	5	↓ TP, albumin, globulin	BUN, Glc., AP, AST, γ-GT, CK, Na, K, Cl, Ca, P	2010 [41]
broilers, 1–21	(15)			
0, 2	5	↓ TP, albumin, globulin, AP, Glc, Ca, P	BUN, AST, γ-GT, CK, uric acid, Na, K, Cl	2010 [41]
broilers, 1–21	(15)			
0, 3	20	↓ TP, ALT	-	2001 [42]
Hubb, 1–21		↑ AST		
0, 3.5	6	↓ TP, albumin, Chl, creatinine, Ca, MCV	AP, ALT, P, RBC, MCH, MCHC	1997 [45]
broilers, 1–21	(18)			
0, 4	6	↓ TP, BUN, Chl, PMCV, hematocrit %	-	1997 [43]
♂PetxHubb, 1–21	(12)			
0, 4	5	↓ TP, albumin, globulin, Chl, Glc., Ca, P	-	1998 [46]
♂broilers, 1–21	(15)	↑ Na, Cl		
0, 5	6	↓ TP, albumin, Chl, uric acid, AP, Ca.	P	1998 [56]
AAxPet, 1–21	(12)	↑ CK		
0, 5	6	↓ TP, albumin, Chl	-	1998 [45]
broilers, 1–21	(12)	↑ BUN, CK		

* Number of replicates. The figure in parenthesis indicates number of animals per replicate. Abbrev.: AA, Arbor Acres; ALT, alanine transferase; AP, alkaline phosphatase; AST, aspartate amino transferase; BUN, blood urea nitrogen; Chl, cholesterol; CK, creatinine kinase; conc., concentration; Glc, glucose; Hb, hemoglobin; Hubb, Hubbard; Pet, Peterson; MCV, mean corpuscular volume; MCH, mean corpuscular hemoglobin; MCHC, mean corpuscular hemoglobin concentration; RBC, red blood cell; γ-GT, γ-glutamyl transferase.

## 4. Effects of Aflatoxin B_1_ on Adaptive Immunity

Secondary to the effects on liver, the immunosupressive nature of AFB_1_ is the best documented area of its toxicity. Recent epidemiological data also indicate high correlation between outbreaks of Newcastle disease (ND) and aflatoxin contamination of broiler rations [57,58].

Generally, the immunotoxic dose of AFB_1_ is considered as less than the dose required eliciting a reduction in bird performance. Selected studies on the effects of AFB_1_ on response from vaccines (humoral immunity), and cell mediated immunity are presented in Table 4. Though several contradictory reports are available, the threshold dose of AFB_1_ may be generalized to be 0.4 and 1 mg/kg for the negative effects on cell mediated and humoral immunity, respectively. However, the question regarding susceptibility of modern broiler regarding immunotoxicity remains yet to be answered. Furthermore, there is evidence regarding biphasic nature of the effects of AFB_1_ on humoral immunity. In this regard our recent data (Table 5) indicate that humoral immune response from broilers could increase and decrease depending upon the level and length of exposure to the toxin. 

**Table 4:** Effects of AFB_1_ on humoral and cell mediated immunity in chicken.

AFB_1_ Level (ppm)	Vaccine Age	Effects	No Effects	Year of Study and Reference
Bird Type, Age (days)				
*Humoral immunity*:				
0.1, 0.2, 0.4, 0.5, 1.0	?	-	Titers to ND and fowl cholera	1985 [59]
Broiler, 14–49				
0.1, 0.2, 0.4, 0.8	?	-	Titers to ND and fowl cholera	1985 [60]
broiler, 14-–49				
1 (AF)	14 days	↓ ND titers at 1 and 3 weeks post vaccination	ND titers at 2, 4, and 5 weeks post vaccination	2003 [61]
Broiler, 7–49				
2.5 (AF)	7 + 21 days	↓ ND titers at 28 days age	-	2000 [62]
Faobro, 1–21				
0, 0.6, 1.2, 2.5	-	↓ total complement activity at 2.5 ppm	total complement activity at 0.6 and 1.2 ppm	1985 [63]
Broiler, 1–42				
5	1 + 21 days	↑ secondary antibodies against IBD at 28 and 35 days	-	1997 [64]
Broiler, 1–35				
0.2	?	↓ antibody titers to ND, IB, and IBD	-	1998 [65]
♀Leghorn, 126–280				
2.5	21 days	-	ND, IB titers; at 35 days susceptibility to ND	1978 [66]
♂Leghorn, 1–28				
2.5	21 days	-	ND titers; susceptibility to ND at 35 days	1978 [66]
♂Leghorn, 1–49				
*Cell mediated immunity*:				
0, 0.1, 0.2, 0.4, 0.8	-	↓ DHST from 0.2 ppm	-	1985 [60]
Broiler, 14-49				
0.1, 0.2, 0.4, 0.5, 1.0	-	↓DHST at 0.4 ppm AFB_1_ + AFB_2_	DHST on AFB_1_ alone	1985 [59]
Broiler, 14–49				
1	-	↓ DHST	-	2003 [61]
Broiler, 7–49				
0.3	-	↓ DHST at 30, 45, and 60 days age	-	1988 [67]
Leghorn, 1–42				

Abbrev.: ↓ reduction; ↑ increase; ? not specified; - data not relevant; DHST, delayed hypersensitivity skin test; IB, infectious bronchitis; IBD, infectious bursal disease; ND, Newcastle disease.

The data presented in Table 5 are not the first observations of increase in humoral immune response during initial stages of exposure to low levels of AFB_1_. Similar results, *i.e.*, an initial increase followed by a decrease in humoral immune response, have been documented in at least two previous reports. However, these effects of AFB_1_ were not discussed in any of these reports and thus had remained overlooked. For instance, Giambrone *et al.* [59] who conducted two separate experiments in 1985 on Hubbard broilers, noted a non-significant increasing trend in ND titers with increase in the AFB_1_ content of ration from zero to 0.5 mg/kg in one of these experiments. Also, a higher (*P* < 0.05) response from fowl cholera vaccine was noted in the birds fed 0.5 mg AFB_1_/kg diet. In the other experiment, higher (*P* < 0.05) ND, and fowl cholera titers were noted in birds fed 0.1 mg, and 0.2 mg AFB_1_/kg diet, respectively. The increase in titers against ND and fowl cholera in birds fed on AFB_1_ contaminated ration was not seen in the birds fed on rations containing mixtures of AFB_1_ and AFB_2_. In a latter study, these authors reported non-significantly higher titers against ND and fowl cholera in birds fed on 0.1 to 0.8 mg AFB_1_/kg rations as compared to the birds fed on control ration [60]. The underlying mechanisms for this temporary increase in humoral immune response are not known. As a matter of fact, the exact mechanisms of even immunosuppression during aflatoxicosis are not clearly understood in spite of 50 years of research on the mycotoxin. In this regard, Corrier [68], and Surai and Dvorska [54] have reviewed some aspects of AFB_1_-induced immunotoxicity. A brief but comprehensive discussion on the subject can also be found in an article by Celik *et al.* [49]. It is a general observation that size of lymphoid organs is not normal in birds exposed to AFB_1_ (Table 2). In such animals, lymphoid cell depletion in thymus, spleen, and bursa of Fabricius has been described [61]. Thus one explanation of immunotoxicity of AFB_1_, as also proposed by Azzam and Gabal [65,69], could be inhibition of antibody production through the toxin’s effects on lymphocytes leading to enhanced turnover of serum antibodies and consequently to decreased antibody half-life.

**Table 5:** Effects of level and length of AFB_1_ exposure on ELISA titers against Newcastle disease and serum protein in Ross 308 broilers ^1^.

Item	2nd Week Exposure	4th Week Exposure	5th Week Exposure
*Titers against ND*:			
0.07 mg AFB_1_/kg diet	33%	407%	−27%
0.75 mg AFB_1_/kg diet	127% *	594%	−28%
*Serum protein*:			
0.07 mg AFB_1_/kg diet	5%	−2%	2.6%
0.75 mg AFB_1_/kg diet	−32% **	−32% *	−21% *

Significant differences with regards to control with * at *P* < 0.05, ** at *P* < 0.01. Data presented as percentage change over control. ^1^ Experiment conducted in 2010 (author’s unpublished data). Statistical analysis by using ANOVA and LSD (*n* = 7/treatment).

During earlier studies on effects of AFB_1_, Tung *et al.* [70] described the toxin-induced increase in lysosomal enzyme activity in liver and skeletal muscles of chicken. These authors postulated that this increase in lysosomal activity, besides other factors, could negatively affect tissue integrity during aflatoxicosis. In this regard, dietary AFB_1_ has been found by Çelik *et al.* [49] to result in degeneration of follicle associated epithelium (FAE) in bursa of Fabricius and destruction of thymic cortex in chicken. On the grounds of the report of Tung *et al.*, it was therefore urged that any impaired function of FAE might result in serious deficiencies in both cellular and antibody responsiveness of the chicken immune system [49]. This is because FAE of bursal follicles play a crucial role in antigen presentation to the lymphoid cell population. Besides the effects on lymphocytes, non-specific effects of the toxin on protein synthesis through inhibition of RNA polymerase, lipid peroxidation, and liver injury are also considered to result in reduced immunoglobulin production. The data presented in Table 5 however indicate modulation of serum protein and antibody titers in different directions. This indicates that AFB_1_-induced modulation of humoral immunity in broilers may not be a result of the toxin’s non-specific effects on protein metabolism.

## 5. Effects of Aflatoxin B_1_ on Gastrointestinal Tract

Gastrointestinal tract is the main site where conversion and absorption of food components takes place. The host-derived physiological processes, the residing microorganisms, and healthy absorptive surfaces are all equally important to ensure normal nutrient supply. Gastrointestinal tract is the first organ coming into contact with mycotoxins of dietary origin and should be expected to be affected by AFB_1_ with greater potency as compared to other organs. However, this aspect of aflatoxicosis is the often neglected area of mycotoxin research and available literature is non-conclusive. 

### 5.1. Aflatoxin B_1_ and Gut Morphology

Various studies documenting effects of AFB_1_ on weight and histological characteristics of different segments of GIT are summarized in Table 6. The weights of proventriculus, gizzard, and pancreas relative to body weight of broilers have not been reported to be affected at levels of AFB_1_ up to 3.5 mg/kg diet [39,44,48]. However, at a dietary level of 4 mg AFB_1_/kg or higher, the relative weight of these organs has been noted to decrease by some authors [45,56], while increase by other [46]. However, Edrington *et al.* could not find any effect of 4 mg AFB_1_/kg diet on the relative weight of gizzard and pancreas [43].

Literature on the effects of AFB_1_ on histology of GIT is scanty and not conclusive. In this regard, the density of whole intestine (weight/length) has been reported to decrease after 3 weeks of dietary exposure to AFB_1_ at levels as low as 0.02 mg/kg [48] and 0.7 mg/kg [71]. As the width of muscularis tends of be relatively constant, the density of intestine could be a good indicator of unit absorptive area. On this variable, the effects of higher AFB_1_ dosage in broilers are not known. At higher levels of 1 mg AFB_1_/kg diet, Kumar and Balachandran however noted catarrhal enteritis with lymphocytic or mononuclear cell infiltrations in the intestine of broilers fed on the toxin contaminated ration for 4 weeks [72]. Contrary to these reports, no histopathological changes in duodenum, jejunum, cecum, and ileum could be noted by Ledoux *et al.* when male broilers were exposed to 4 mg AFB_1_/kg diet for 3 weeks [46]. Similarly, breaking strength, size, and collagen content of large intestine was not found to be affected in broilers (male Cobb × Cobb; exposure age 1 to 21 days) exposed to 0, 0.6, 1.2, 2.5, 5.0, and 10 mg AFB_1_/kg diet in the earlier report by Warren and Hamilton [73]. Lipid content of large intestine was decreased only at the highest level of AFB_1_ (10 mg/kg) in that report.

From the aforementioned studies, it is difficult to draw a dose-effect relationship between AFB_1_ and histological changes in the GIT. This is because specific sections of GIT, studied variables, and length of exposure were different in the aforementioned studies. Furthermore, the type and specific line of chicken used in various studies may also affect the reaction of intestine towards chronic aflatoxicosis. This hypothesis is supported by the recent observations regarding aflatoxicosis in layers (Hyline W36; exposure age from 140 to 154 days) by Applegate *et al.* [74]. Contrary to the observations in broilers, these authors noted a linear increase in the crypt depth in distal jejunum with the increasing levels of AFB_1_ in the diet as 0, 0.6, 1.2, and 2.5 mg/kg, but no effect of the toxin on villus height and number of goblet cells. However, the duration of exposure to AFB_1_ was short as compared with other studies and may not be long enough to provoke morphological changes in jejunum of layers.

**Table 6:** Weight and histology of individual segments of gut in chicken during exposure to AFB_1_.

AFB_1_ Level (ppm)	*n* *	Characteristics of Gut		Year of Study and Reference
Bird Type, Age (days)		Effects	No Effects	
0.07, 0.7	(7)	↓ Density of duodenum and jejunum	Weight of proventriculus and gizzard	2011 [71]
♂ Ross308, 7–29		↑ Length of duodenum and jejunum		
0.02	5	↓ Density of intestine	Gizzard weight; intestinal weight and length	2010 [48]
♂ Hybro, 21–49				
0.1	4	-	Pancreas weight	2010 [57]
♂ Ross308, 427–457	(3)			
0.3	12	-	Gizzard weight	2000 [39]
Broilers, 1–35				
1	2	Necrosis/fibrosis in crop and proventriculus. Catarrhal enteritis in intestine	-	2009 [72]
Broiler, 1–28	(5)			
0.6, 1.2, 2.5	8	linear effect: ↑ crypt length in distal jejunum	Number and density of goblet cell in jejunum	2009 [74]
♀ W36, 140–154				
3.5	6	-	Gizzard weight	1997 [44]
Broilers, 1–21	(4)			
4	6	-	Gizzard and pancreas weight	1997 [43]
♂PetxHubb, 1–21	(3)			
4	5	↑ Proventriculus and pancreas weight	Microscopic evaluation of pancreas and whole GIT	1998 [46]
♂ Broilers, 1–21	(3)			
5	6	↑ Gizzard and pancreas weight	Proventriculus weight	1998 [56]
AA x Pet, 1–21	(2)			
5	6	↑ Proventriculus and pancreas weight	-	1998 [45]
Broilers, 1–21	(2)			
0, 0.6, 1.2, 2.5, 5, 10	4	-	Breaking strength and size of large intestine	1980 [73]
♂CobbxCobb, 1–21	(10)			

* Number of replicates. The figure in parenthesis indicates number of animals per replicate. AA, Arbor Acres; Hubb, Hubbard; Pet, Peterson; W36, Hyline W36.

From the recent studies of Kana *et al.* [48], Yunus *et al.* [71], and Kumar and Balachandran [72] in broilers, it appears that the unit absorptive surface of small intestine would deteriorate during a chronic exposure to low levels of AFB_1_. However, broilers have been noted to compensate the reduced unit absorptive surface by increasing the length of small intestine in one study [71]. Such a reaction of intestinal tissues to low levels of AFB_1_, if also proven in future studies, would certainly add to the present understanding regarding intestinal adaptability to chronic AFB_1_ exposure.

### 5.2. Aflatoxin B_1_ and Active Transport of Nutrients

After a thorough search of various databases only two reports could be found in which the issue of intestinal active transport of nutrients during aflatoxicosis was addressed. In this connection Ruff and Wyatt showed that 3 weeks feeding of 1.25 to 5 mg AFB_1_/kg diet has no effect on *in vitro* absorption of glucose and methionine in the intestine of broilers [75]. However, a high dose of 10 mg AFB_1_/kg diet, for more than 1 week, increased both the mediated and diffusion components of glucose and methionine absorption. Absorption of glucose and methionine was not affected in broilers exposed to these high amounts of AFB_1_ for only one week in the study of Ruff and Wyatt. In the second study which utilized murine *in vitro* model, acute exposure to AFB_1_ (5 µg/mL of buffer) was not found to affect glucose uptake in everted rat jejunum [76].

In some studies, active nutrient uptake was not addressed but movement of ions across intestinal epithelia and activity of ion transporters was studied. These studies may give some insight into the possible mechanisms of effects of AFB_1_ on active transport of nutrients including glucose absorption. This is because the active absorption of glucose through sodium glucose co-transporter (SGLT1) is influenced by intracellular levels of Na^+^ and movement of other ions across a cell. In this regard, Chotinski *et al.* [77] studied the effects of 7 weeks of dietary exposure to 0.25 and 0.6 mg AFB_1_/kg diet on activity of Mg^2+^(Na^+^/K^+^)-ATP in small intestinal mucosa of broilers. In this study, 0.6 mg AFB_1_/kg diet was found to suppress the activity of Mg^2+^(Na^+^/K^+^)-ATP in small intestinal mucosa. Recently, acute AFB_1_ exposure has been reported to evoke acetylcholine-sensitive contractions in the rat ileum [78]. One effect of acetylcholine and other cholinergic secretagogues is to increase basolateral K^+^ efflux and apical Cl^−^ secretion in epithelia [79]. During higher outgo of anions from epithelia, a lower absorption of Na^+^ and consequently lower absorption of glucose is expected. In this regard *in vitro* AFB_1_ has been found to evoke cholinergic secretion of Cl^−^ and negatively affect glucose absorption in broiler’s jejunum [80]. However, these effects of acute exposure could not be established for a chronic exposure of broiler’s to low levels of AFB_1_ [71]. It therefore seems that intestinal tissues may adapt to an on-going dietary challenge to low levels of AFB_1_ as far as active transport of nutrients is concerned.

### 5.3. Aflatoxin B_1_, and Digestibility and Activity of Digestive Enzymes

Aflatoxin B_1_ is widely believed to result in malabsorption syndrome regarding macro nutrients and also in reduced activity of digestive enzymes [38,54]. However, many reports contrary to this notion are available. For instance, Nelson *et al*. did not find any effect of AFB_1_ (natural contamination of corn with *A. flavus*) on dry matter (DM), and amino acid digestibility, and energy utilization in chicken [81]. Applegate * et al*. did not find any effect of 0.6, 1.2, and 2.5 mg AFB_1_/kg diet on digestibility of DM and nitrogen (N) per hen/day [74]. At 0.6 and 1.2 mg AFB_1_/kg diet, the apparent metabolizable energy (AME) was however found to be reduced in their study. Regarding the activity of pancreatic enzymes, Mathur * et al.* found higher amylase and chymotrypsin activity, while lower lipase activity after exposure of Ross 308 female birds to 0.1 mg AFB_1_/kg diet (at 427 to 457 days age) [55]. The activity of trypsin in pancreas was not affected by AFB_1_ treatment. These results, except for reduction in lipase activity, are supported by earlier work of Richardson and Hamilton on layers [82]. These authors reported that 4 mg AFB_1_/kg diet increases the activity of pancreatic chymotrypsin, amylase, and lipase. Pancreatic trypsin was not affected by AFB_1_ in their study and the noted changes in the pancreatic secretions were also not reflected in the lipid content of the feces. Contrary to these two reports, Osborne and Hamilton noted lower activity of pancreatic amylase, trypsin, lipase, RNase, and DNase when broilers were exposed to 1.25 and 2.5 mg AFB_1_/kg diet [83].

Regarding activity of intestinal enzymes, Mathur *et al.* in their aforementioned report found lower lipase activity in duodenum after exposure of Ross 308 female birds to 0.1 mg AFB_1_/kg diet [55]. These authors found that AFB_1_ at the tested low level had no effect on amylase and chymotrypsin activity in duodenum and jejunum, and lipase activity in jejunum. The activity of trypsin in duodenum, and jejunum was also not affected by the AFB_1_ treatment. Contrary to the case of broilers, Applegate *et al.* found the intestinal maltase activity to increase quadratically up to the doses of 1.2 mg AFB_1_ while decrease at 2.5 mg AFB_1_ with the exposure of layers (from 140 to 154 days age) to 0, 0.6, 1.2, and 2.5 mg AFB_1_/kg diet [74]. These changes were however not found to affect digestibility and retention of DM and N.

From the presented literature, it is impractical to draw any conclusions regarding effects of a certain level of AFB_1_ on digestive functionality in broilers. Some studies, in the past decade however indicate that the decreased nutrient utilization observed in those studies might be a factor of the effects of the toxin on systemic metabolism rather than an effect on digestive functionality [84,85]. This notion is also supported by the fact that apparent metabolizable energy of AFB_1_-contaminated rations was noted to be negatively affected by all the authors who included this variable in their studies and did not find any effect of the toxin on nutrient digestibility. More studies are no doubt needed in this direction.

### 5.4. Aflatoxin B_1_ and Intestinal Innate Immunity

The innate immune system of intestine plays a vital role in maintaining the integrity of the intestine and also participates with adaptive immune system in ensuring the subtle equilibrium between immune tolerance and immune response in the GIT [86]. Intestinal intraepithelial cells (IEC) in this regard produce a diversity of antimicrobial peptides and enzymes that protect intestinal mucosa and crypts against microbes. Some of these molecules also function in alarming the adaptive immune system. Contrary to many other mycotoxins, AFB_1_ has not been considered to date for possible effects on these peptides and enzymes. Furthermore, the barrier function of IEC during aflatoxicosis has not been subjected to extensive research. This passive barrier is formed by the IECs themselves, the tight junctions sealing the intercellular spaces, and the mucus secreted by them. This barrier provides a passive means to prevent most bacteria and antigens entering the body, and at the same time minimizes electrolyte and fluid loss into the intestinal lumen. Transepithelial electrical resistance (TEER) is an important indicator of barrier function of IEC. Limited data related with intestinal health suggests that AFB_1_ can only moderately affect TEER during acute exposure to the toxin [80]. In the report of Warren and Hamilton, mentioned under Section 5.1, a 3 weeks administration of AFB_1_ at the levels of 0, 0.6, 1.2, 2.5, 5.0, and 10 mg/kg diet to broiler chicks however did not affect the gross variable of breaking strength of large intestine [73].

In murine models, AFB_1_ has been found in some studies to result in morphologically damaged intestinal mucosal linings [87]), and in decreased cell proliferation [88]). In this regard, Watzl *et al.* found AFB_1_ to induce genotoxicity (comet assay) in isolated rat jejunal epithelial cells. However, oral exposure of rats to moderate doses of AFB_1_ (100 µg/kg body weight once a week for 5 consecutive weeks), in the same study, was not found to induce DNA damage in jejunal epithelium [89]. Most recent report in this connection is of García *et al.*, who reported that AFB_1_ acts in synergy with fumonisins in affecting intestinal barrier function as determined by cellular proliferation, cellular damage, and synthesis of IL-8 in porcine intestinal epithelial cell line [90]. Aflatoxin B_1_ alone was found in this report to only affect the morphological characteristics of the cells and not other variables. From reports on species other than chicken, moderate and indirect effects (secondary to systemic) of AFB_1_ on TEER of small intestine may be speculated. The practical significance of any such effect has been a subject of three different studies. In this connection, Rao * et al.* studied the clinical signs and gross lesions caused by *Eimeria uzura* in Japanese quail during intercurrent dietary aflatoxicosis [91,92]. In these studies, no significant differences in the mucosal morphology of the intestine were evident histologically. However, these authors found that the combination of *E. uzura* infection and aflatoxicosis causes reduced packed cell volume and hemoglobin, weight loss, increased coccidian oocyst production, and higher morbidity (60 *vs.* 8.3%) and mortality (28.3 *vs.* 6.6 and 21.6%) as compared to the coccidia or toxin alone. It was concluded that aflatoxicosis may influence the course of coccidial infection due to additive effects. In an earlier study on broiler’s exposure to 2.5 μg AFB_1_ and/or *Eimeria acervulina*, Ruff *et al.* also concluded that the birds exposed to combined treatment gain significantly less weight with greater plasma depigmentation (deduced from plasma β-carotene level), but without apparent differences in gross lesions in intestine caused by the coccidian [93].

### 5.5. Interaction of Aflatoxin B_1_ with Gut Microbes

Since the 1940s various studies have shown antimicrobial potential of several mycotoxins [94,95,96,97,98]. Regarding aflatoxins, the study of Burmeister and Hesseltine in 1966 was probably the first comprehensive study in which several microorganisms (329 spp.) were tested for their sensitivity against AFB_1_ [99]. Among the strains tested in that investigation, 12 species of genus *Bacillus*, a *Streptomyces* sp. and *Clostridium sporogenes* were inhibited when various levels of AFB_1_ (15–30 μg/mL) were incorporated into the growth substrate. None of the yeast strains tested in the study was affected by AFB_1_ even at 40 μg/mL concentration. *Bacillus megaterium* and *B*. *brevis* were most susceptible to AFB_1_, and many of the subsequent studies demonstrated the extreme sensitivity of *B*. *megaterium* to AFB_1_ (as low as 1 μg AFB_1_/mL) [100,101,102]. Contrary to the study of Burmeister and Hesseltine, inhibitory effects of AFB_1_ on many fungal strains including *A. flavus* itself, *A*. *awamori*, *Penicillium chrysogenum*, and *P*. *duclauxi* were reported later [103]. Similarly, 10 ppm AFB_1_ was found to inhibit the enzyme activity of *Mucor hiemalis* [104]. In other contemporary studies *A. niger*, *A*. *parasiticus*, *P. expansum*, *Cladosporium herbarum*, *Rhizopus nigricans*, *Thamnidium elegans*, and *Neurospora crassa* were also identified as being sensitive to 50 to 100 μg AFB_1_/mL [105,106,107,108]. A comprehensive review of these studies has been presented earlier by Reiss [109]. In recent studies, AFB_1_ was found to selectively inhibit *Streptococcus agalactiae*, *S. aureus*, and *Yersinia enterocolitica* [110]. 

Most of the earlier literature was dedicated to finding suitable bacterial test strains for mycotoxin bioassays. However, the biological methods for detection of aflatoxins were found to be of little use in the surveillance of the toxin [111]. Several *E*. *coli*, *Salmonella* typhimurium, and *Bacillus* strains on the other hand have found uses as testers in genotoxic studies [112,113,114,115,116]. Other than the genotoxic effects, the toxic effects of aflatoxin on various microbes have been proposed to be as inhibition of oxygen [117] and inulin uptake [118], generation of oxygen radicals [119] and formaldehyde and its reaction products [120], and damage to cell membrane causing leakage of cell contents [118,121]. An interesting feature of these antimicrobial effects is that during continuous exposure to AFB_1_, some sensitive bacterial species (*B. cereus*, *Proteus mirabilis*) are able to survive the toxic effects to the extent that their growth is enhanced by presence of the mycotoxin [122] indicating ability to metabolize AFB_1_.

In spite of the indicated antimicrobial potential of AFB_1_, data regarding effects of the toxin on gut microbial population and fermentation are scanty. Kubena *et al.* in this regard performed two, 10-days experiments to study cecal VFA’s and broiler chick susceptibility to *Salmonella* typhimurium colonisation as affected by 2.5 and 7.5 mg aflatoxins/kg diet [123]. In one of these experiments no effects of aflatoxins were found on *Salmonella* colonization and on cecal VFA production. However, in the second experiment, both dietary levels of aflatoxins resulted in significant increase in total VFA’s at 5 days age. The lower aflatoxin dose (2.5 mg/kg diet) appeared to be more effective as it also resulted in significantly higher total VFA’s at 7 days age.

Related to the issue of effects of AFB_1_ on gut microbes, interesting data were presented in an earlier study of Larsen *et al.* [124]. These authors studied the effect of AFB_1_ on susceptibility of hamsters to orally administered *Mycobacterium paratuberculosis*. In the negative control group, the bacillus passed the epithelial barrier of the intestine and infection was established in small intestine and mesentric lymph nodes. In the positive control and test groups, aflatoxin-treated hamsters grew slowly and showed signs of AFB_1_ toxicity. Interestingly, the addition of AFB_1_ to the rations did not increase the susceptibility of hamsters to *M. paratuberculosis*, rather it decreased susceptibility to the bacillus. Hamsters not treated with AFB_1_ and infected with *M*. *paratuberculosis* had higher intestinal bacterial counts than did infected hamsters that had been treated with AFB_1_. These results are substantiated by the study of Abdelhamid *et al.* who found that effects of AFB_1_ on rumen fermentation may be like antibiotics: Affecting the harmful flora and encouraging the rumen microflora as noted by slight improvements regarding *in vitro* rumen fermentation of wheat straw and berseem (*Trifolium alexandrinum*) hay after dietary AFB_1_ exposure [125]. In this regard, fermentation patterns of *Saccharomyces cerevisiae*, and several *Lactobacillus* spp. have been noted to change under the influence of AFB_1_ [126,127]. Sutic and Banina in this regard reported that under the influence of AFB_1_, *Lactobacillus casei*, *L*. *plantarum*, and *Streptococcus lactis*, well known as not producing gas from glucose and other sugars, became heterofermentative and started producing significant amount of gas [128]. However, these studies do not warrant any positive effects of AFB_1_ on intestinal microbial population.

## 6. Conclusions

Recent literature documents the negative effects of those low dietary levels of aflatoxins which were previously thought to have no impact on broiler performance. Furthermore, available data indicate that both the level and the length of exposure influence the response of broilers towards chronic aflatoxin challenge. Therefore any attempt to establish dose-effect relationship between dietary aflatoxin level and broiler’s performance would be influenced by these factors. Scanty data also indicate that some variables including bird performance and humoral immunity might improve during initial phases of exposure to aflatoxin.

In spite of 50 years of continuous research on aflatoxins, several areas of aflatoxicosis remain yet to be explored. These areas, as discussed in the present attempt, include comparative hepatic metabolism of aflatoxin, and response of gastrointestinal tract to the toxin. Literature available, regarding effects of the toxin on gastrointestinal tract, is particularly non-conclusive. However, there is evidence that gastrointestinal tract may adapt in some ways to a chronic aflatoxin challenge. As gastrointestinal tract is the first organ coming into contact with dietary aflatoxin challenge, its response toward the toxin may yield interesting data regarding tissue adaptability during chronic aflatoxicosis. 
